# Principles of Surgery

**Published:** 1910-07

**Authors:** 


**Principles of Surgery.** By **N. Senn,** M.D., Late Professor of Surgery, University of Chicago. Fourth edition by **Emanuel J. Senn,** M.D., and **Emanuel Friend,** M.D. Octavo, pp. 718. Illustrated. Philadelphia: F. A. Davis Co. (Cloth, $5.00.)

The fourth edition of this well known work has been revised by Emanuel Senn and Emanuel Friend, and is practically the same as in former editions. A few new subjects have been added. Wright's opsonic work, and Bier's treatment of inflammatory affections are discussed as fully as the subjects seem to warrant. The book has been brought up-to-date by the addition of new studies in the treatment of various diseases. The work is so well known and the reputation of the author so well established during his life, that little more need be said than that the fourth edition is now ready for distribution. J. A. R.

